# Supplementary material for: Characteristics of CD133-Sustained Chemoresistant Cancer Stem-Like Cells in Human Ovarian Carcinoma
Source: Int J Mol Sci. 2020 Sep 4;21(18):6467. doi: 10.3390/ijms21186467 (PMC7554888; doi:10.3390/ijms21186467)
Supplement: Supplementary file 1 [file ijms-21-06467-s001.pdf]

International Journal of
Molecular Sciences

MDPI

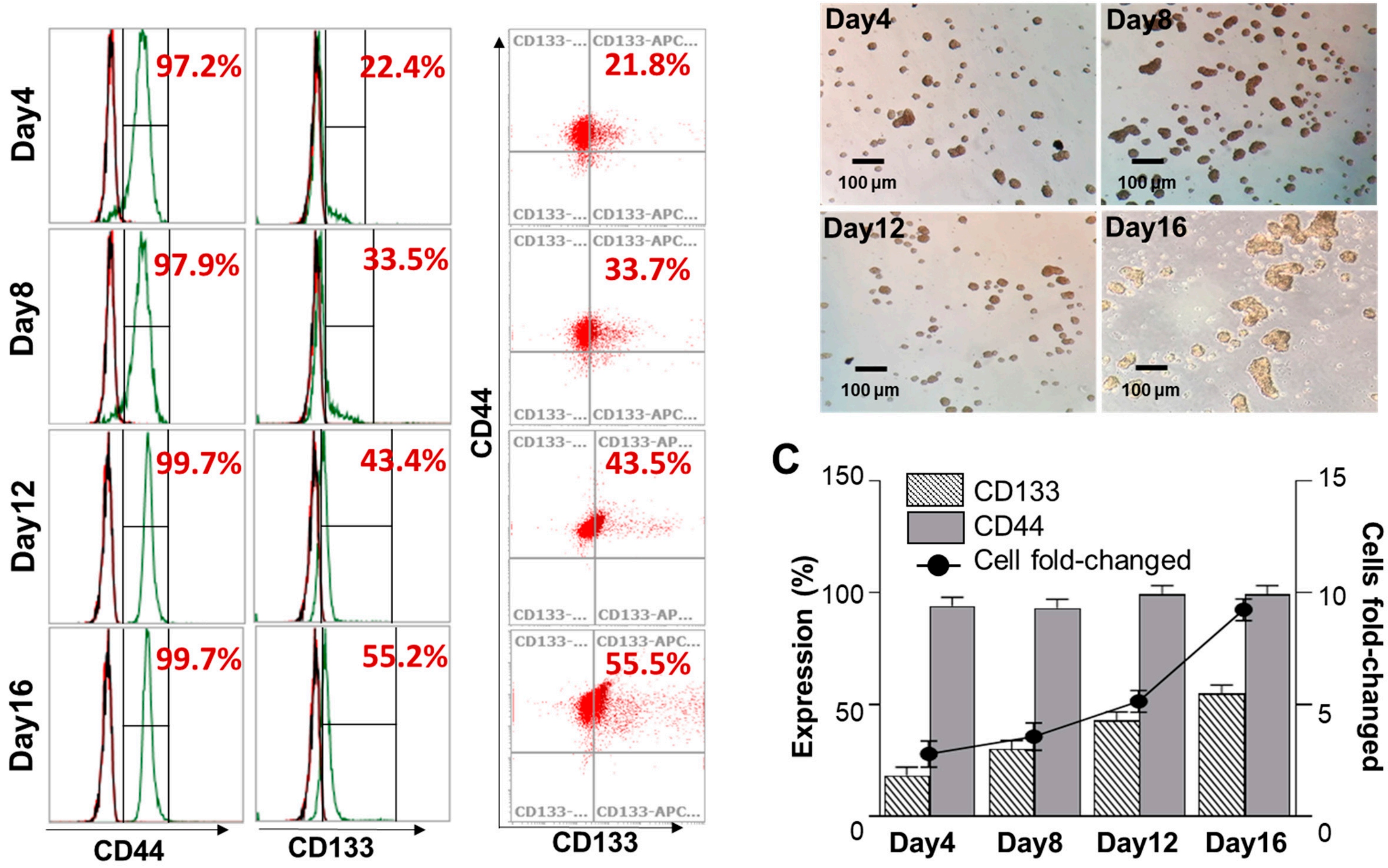

Figure S1. Morphological changes and stemness markers expression of OC-sphere cells derived from SKOV3 cells. OC-sphere cells derived from human SKOV3 cells at days 4, 8, 12, and 16. SKOV3-sphere cells were collected at each time point for (**A**) cell surface CD44 and CD133 expression assessed by flow cytometric analysis, (**B**) morphological changes using optical microscopy imaging, and (**C**) cell-fold change correlated with CD133 expressions during sphere-induction period.

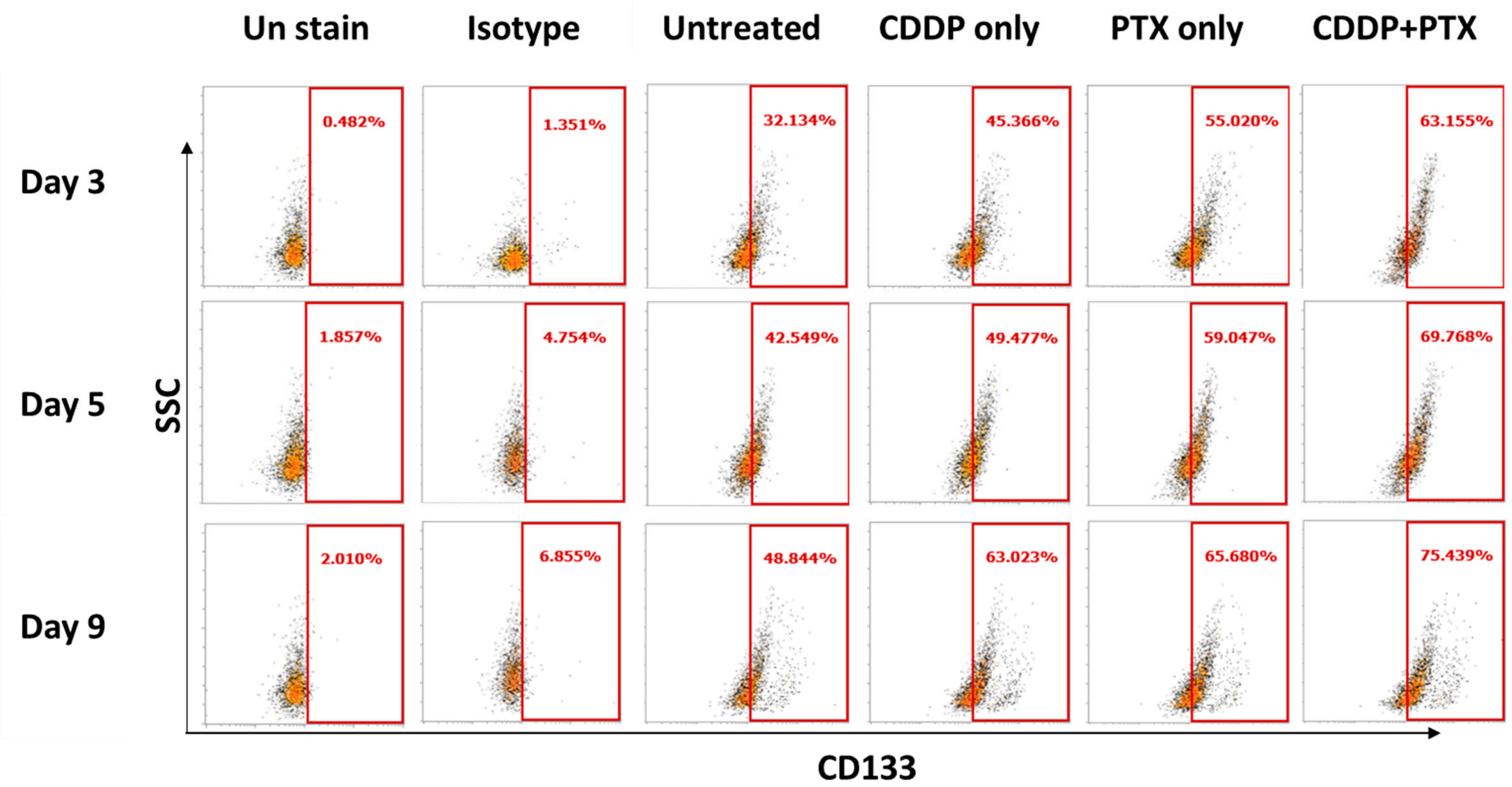

Figure S2. CD133 expression of OC-sphere cells derived from drugs pretreated-SKOV3 cells. A day 4 induction of OC-sphere cells derived from human SKOV3 cells pretreated with 1 μM CDDP alone, 1 nM PTX alone, and CDDP+PTX combination for days 3, 5, and 9. Represented dot-plot results were cell surface CD133 expression assessed by flow cytometric analysis.

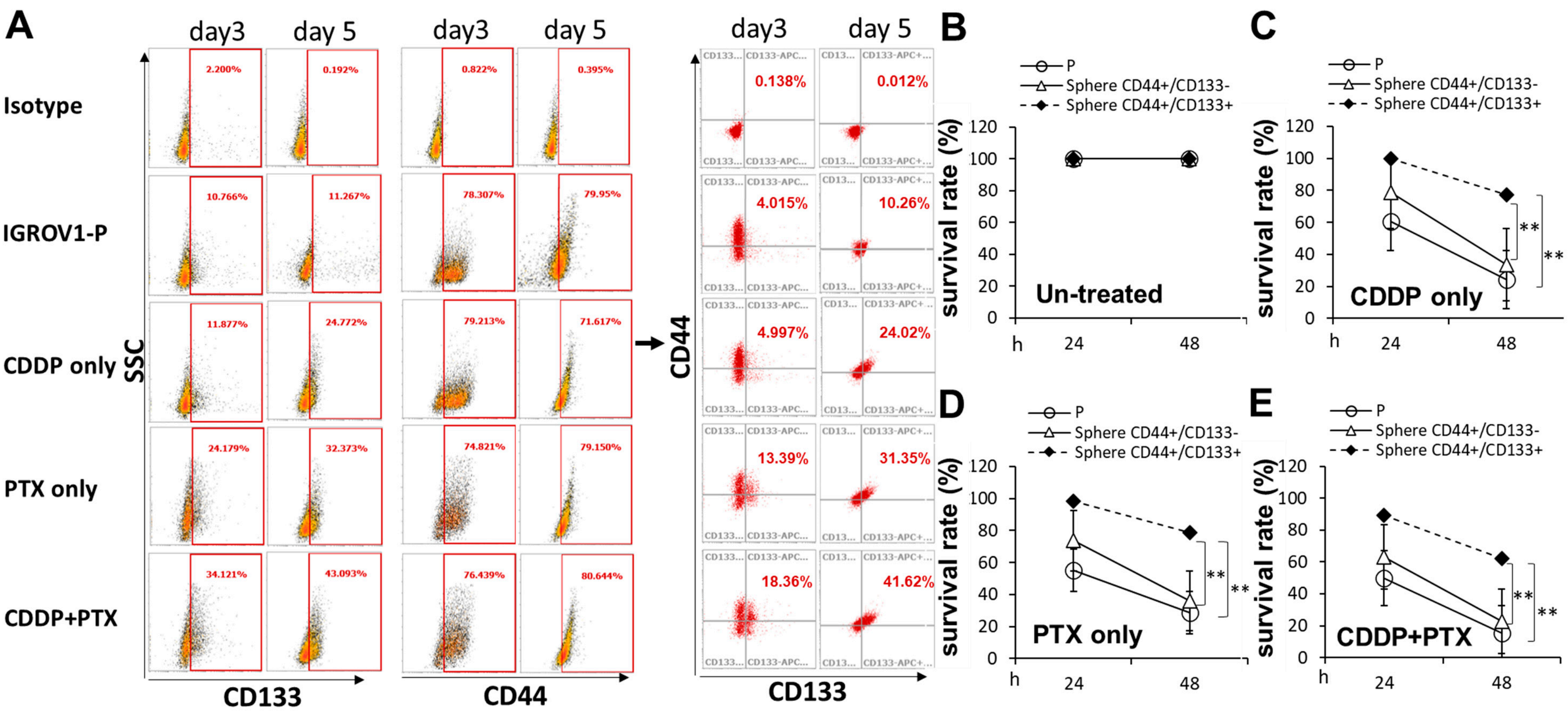

Figure S3. Pretreatment with cisplatin (CDDP) combined with paclitaxel (PTX) synergistically enhanced the sphere-forming ability, cluster of differentiation 133 (CD133) expression, and chemoresistant capacities of cancer stem cell (CSC)-like IGROV1 spheres. IGROV1 parental cells (IGROV1-P) were pretreated with 1 uM CDDP alone, 1 nM PTX alone, or their combination for 3 and 5 days, and then sphere-forming and cell surface CD133/CD44 expression assays following a 4-days induction for CD44+CD133+ spheres and CD44+CD133- spheres populations sorted, collected, and analyzed in (**A**) cell viability assays comparisons among untreated in (**B**) CDDP-treated in (**C**) PTX-treated in (**D**) and CDDP/PTX-treated in (**E**) following 24 and 48 h of treatment. Presented data were acquired from three independent experiments. ** $p < 0.01$.

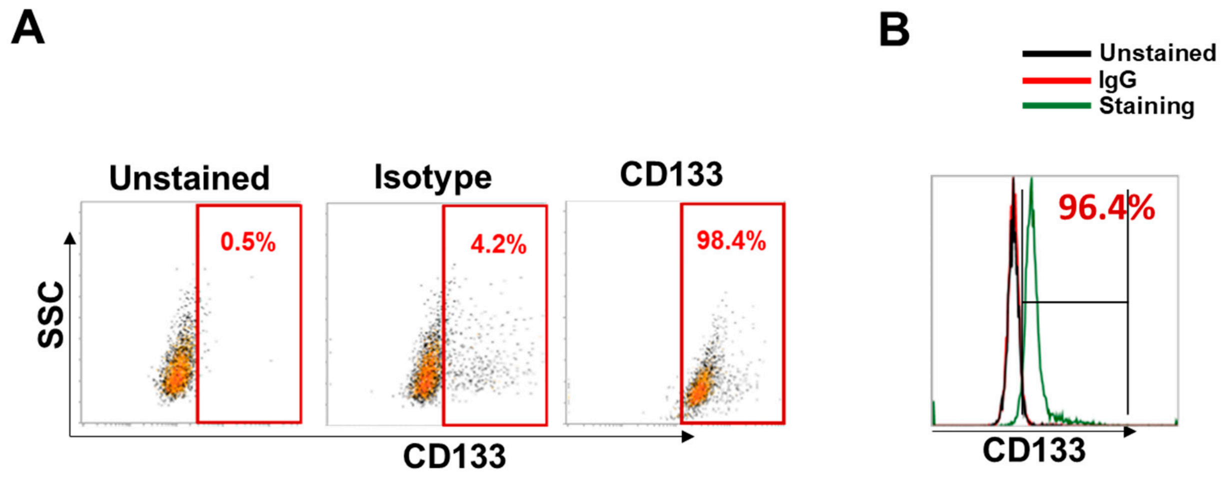

Figure S4. CD133 transduction in human SKOV3 cells. (**A**) and (**B**) CD133 transduction efficiencies were validated by flow cytometric analysis.

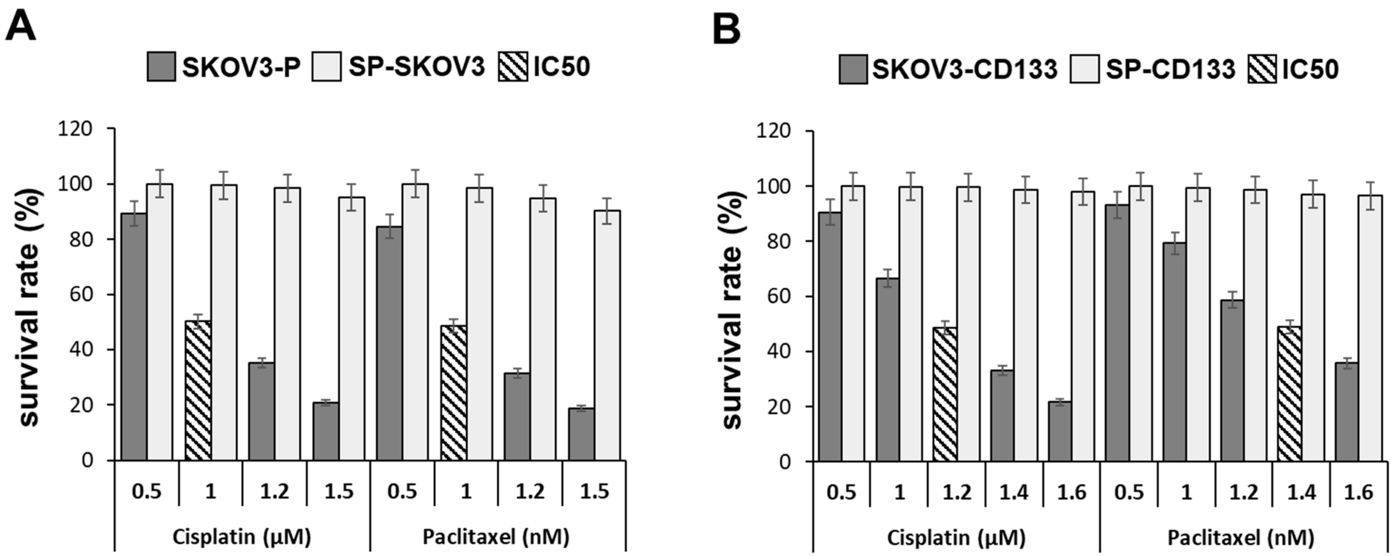

Figure S5. Growth inhibition of cisplatin (CDDP) and paclitaxel (PTX) in SKOV3 parental cells (SKOV3-P) and SKOV3 cells transduced with CD133 (SKOV3-CD133). Both CDDP and PTX were tested in the concentration ranges of 0.5–1.6 μM and 0.5–1.6 nM, respectively. The growth inhibition effect was considered significant when the inhibition rate was around 50% (IC50). The IC50 of CDDP and PTX in (**A**) SKOV3-P and (**B**) SKOV3-CD133, respectively, are shown by slashed bars. Data shown were acquired from three independent experiments.
